# Two-level fixation with headless compression screws for tibial plateau fractures

**DOI:** 10.1007/s00068-022-01982-3

**Published:** 2022-05-14

**Authors:** Robert Kaspar Wagner, Peter Kloen

**Affiliations:** 1 Department of Orthopaedic Surgery, Amsterdam UMC Location University of Amsterdam, Meibergdreef 9, Amsterdam, The Netherlands; 2 Amsterdam Movement Sciences, Musculoskeletal Health, Amsterdam, The Netherlands

**Keywords:** Tibial, Plateau, Fracture, Reduction, Screw

## Abstract

**Purpose:**

Reduction and fixation of tibial plateau fractures associated with small, “floating” intra-articular fragments proposes a challenge. We use fully threaded headless compression screws for (interfragmentary) fixation of such fragments before final plate fixation when standard fixation of intra-articular fragments with k-wires or lag screws is deemed insufficient. Our aim is to describe our technique and clinical experience of this two-level fixation.

**Methods:**

Between 2006 and 2021, 29 patients with a comminuted tibial plateau fracture were treated with this two-level fixation in this retrospective case series. Clinical baseline and surgical variables were collected for all patients. Clinical outcome variables were available for 28 patients with a median follow-up of 16.5 months (IQR 5–24). Functional outcomes were measured with the Knee Injury and Osteoarthritis Outcome Score (KOOS) and reported by 22 patients at a median of 5.2 years (IQR 3.5–9.8).

**Results:**

Reduction was anatomic or good in 82% of cases, fair in 14%, and a malreduction in 4%. Arthrosis was graded as grade 0 in 25% of cases, 1 in 39%, 2 in 21%, and 3 in 14%. Flexion was 110 degrees (IQR 100–130). Five patients had an extension deficit of 5 to 10 degrees. Median KOOS for symptoms and stiffness was 69 points (IQR 45–78), for pain 71 (IQR 45–88), for ADL 85 (IQR 52–95), for sports 30 (IQR 11–55), and for quality of life 34 (IQR 19–56).

**Conclusion:**

The use of fully threaded headless compression screws is a simple and helpful addition in the treatment of comminuted tibial plateau fractures.

## Background

Fractures of the tibial plateau range from a simple non-displaced split to a severely comminuted fracture involving both medial and lateral plateau [1]. Treatment most often consists of open reduction and internal fixation (ORIF), during which the plateau is reconstructed using a step-by-step approach [2, 3]. These steps include a surgical approach that allows for elevation and repositioning of (impacted) fragments and temporary fixation with k-wires that are replaced with definitive osteo-synthesis using screws and plate(s). Metaphyseal defects are often filled with bone graft (substitutes) [3]. This strategy of anatomic reduction and stable fixation using a soft tissue preserving technique that allows early motion remains the cornerstone of treatment. However, there have been some advances in this area: (1) introduction of the three-column concept may allow better pre-op planning [4]; (2) new posterior-based surgical approaches allow for better access [3, 5]; and (3) plates are nowadays smaller and anatomic [6], and allow for a rafting technique giving better support of the subchondral bone.

Despite these advances, reduction and fixation of multiple “floating” small intra-articular fragments can still propose a challenge. If they are not fixated into a stable construct, they will likely displace in the early post-operative period. Reconstruction of these multiple small fragments into one large fragment on the side table or fixation of a small fragment against a “standing” part of the tibial plateau can be helpful in obtaining the perfect reduction. As these fragments may not have a cortical border, the use of standard lag screws can be insufficient. Giannoudis et al. used a “two-level” reconstruction technique to treat comminuted and displaced posterior wall fractures of the acetabulum and achieved good results [7]. They used 1.5 or 2 mm fully threaded cortical mini-screws to maintain anatomic reduction of osteochondral fragments and then reduced and fixated the overlying posterior wall fragment with 3.5 mm lag screws and a buttress plate. Triggered by their idea, we started using fully threaded headless cannulated compression screws (HCS) for fixation of intra-articular fragments in tibial plateau fractures.

The primary aim of the present report is to describe our technique and clinical experience of using HCS for (interfragmentary) fixation of intra-articular fragments in tibial plateau fractures.

## Materials and methods

Between July 2002 and February 2021, 210 tibial plateau fractures were operated on by the senior author in our level 1 Trauma Center. In this group, we identified all patients that were treated with osteo-synthesis including at least one HCS using a prospectively maintained surgical logbook. After ethical approval was waived by the local Medical Ethics Review Committee (W21_182 # 21.197), we retrospectively reviewed the electronic medical records to check radiographs and operative notes to confirm the implantation of the HCS. The study was carried out in accordance with the World Medical Association Declaration of Helsinki. A total of 29 patients were identified and included in this series; the first was operated in 2006 and the last in February 2021.

Clinical baseline and surgical variables were collected for all patients from the electronic medical records. Clinical outcomes were available for 28/29 (97%) of patients. For one patient, clinical outcomes were not available due to foreign residency. The median clinical follow-up duration was 16.5 months (IQR 5–24) after surgery.

In addition, all patients were invited by phone to complete an electronic questionnaire on functional outcome measures and report on total knee arthroplasty (TKA). One patient had passed away and one patient could not participate due to medical reasons. Of the remaining patients, 22/26 (85%) responded at an average of 5.2 years (IQR 3.5–9.8) after surgery. The non-respondents could not be reached using the contact information that was available to us.

### Operative technique

All patients received a pre-operative CT scan. The surgical approach was determined by the fracture pattern. In 14/29 cases, a single-incision approach was used: either anterolateral (*n* = 11), medial (*n* = 2), or posterior (*n* = 1). In 15/29 cases, a 2-incision approach was used: anterolateral/posteromedial (*n* = 8), anterolateral/posterior (*n* = 1), or anterolateral/medial (*n* = 6). Three patients were treated in a staged fashion with a posterior approach, followed by an anterolateral approach a few days later because of swelling. For the lateral plateau, the knee joint was routinely spanned with a large AO femoral distractor for improved visualization. A sub-meniscal arthrotomy was performed for access to the lateral plateau. An osteotomy of the fibular head was added if extensile exposure was necessary (*n* = 2). In bicondylar fractures, the medial plateau was reduced first. For the lateral plateau, the anterolateral fragment was rotated externally, providing access to the metaphyseal impaction. Fragments were reduced and temporarily stabilized with k-wires and clamps under direct vision and fluoroscopic control.

**Fig. 1:**
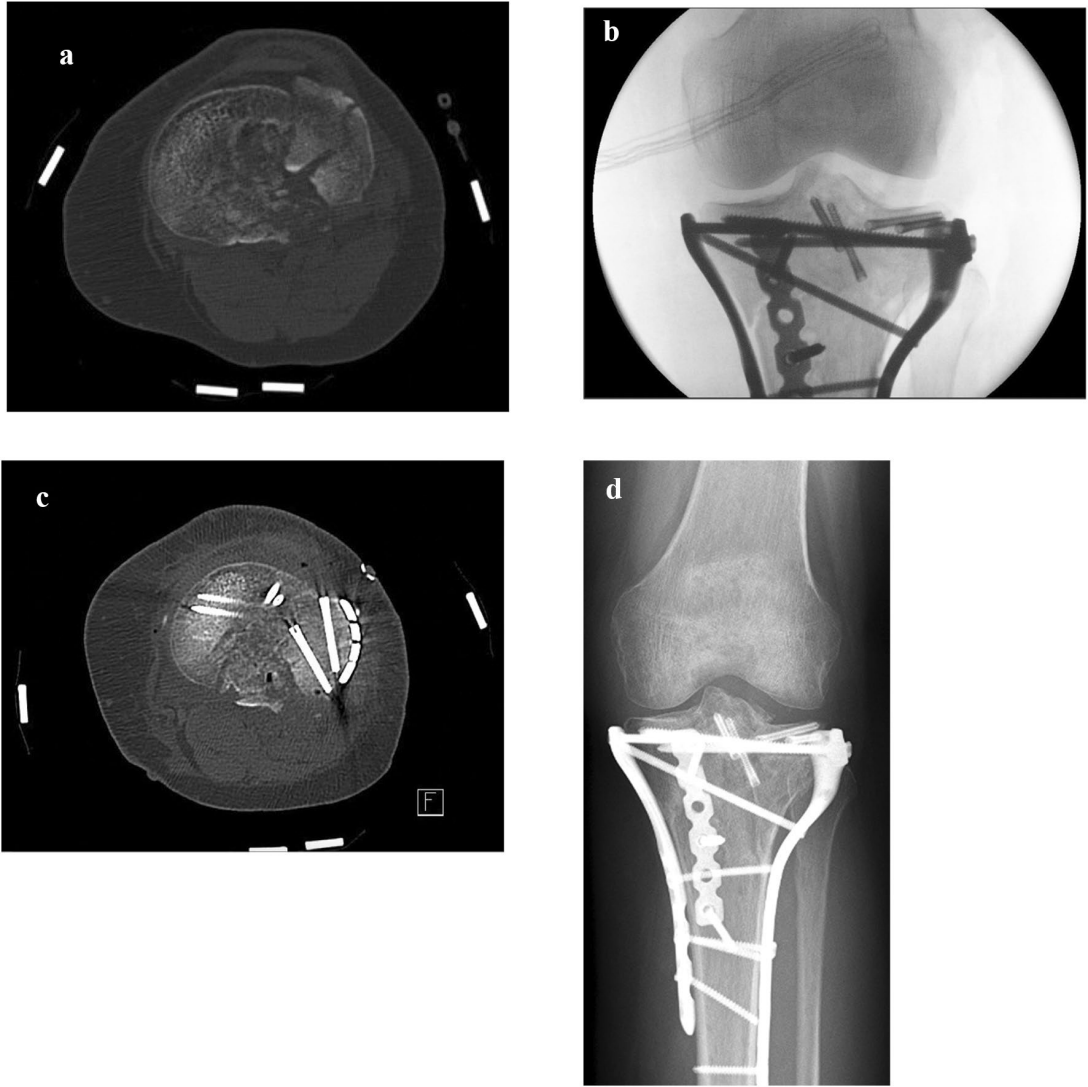
Example of inter-fragmentary fixation in a 52-year-old female with a Schatzker type V fracture of the left knee with two lateral fragments (patient 26). **A** Pre-operative axial CT-scan showing the two lateral fragments. **B** Intra-operative fluoroscopy displaying the HCS used for inter-fragmentary fixation of the lateral fragments and their placement as close to the surface as possible. Two HCS are used to fixate the tibial eminence. **C** Post-operative axial CT-scan showing the concept of “two-level” interfragmentary fixation, where the fused fragment is reduced and fixated with an overlying plate with rafting screws. **D** Radiograph 10 months after treatment with the HCS

When multiple, adjacent intra-articular fragments were present that could not be sufficiently reduced with the use of lag screws (due to the absence of a cortical border), k-wires (due to insufficient compression), or that needed support complementary to the rafting screw construct (due to an inadequate purchase of bone stock of the fragment), inter-fragmentary fixation was performed using one or more (maximum 3) 20–30 mm HCS (Acutrak 2 mini screw; Acumed, Herzele, Belgium). Fragments were either extracted from the fracture site and then fixated on a side table under compression and direct visual control or fixated while maintained at the fracture site. The position of the screws was as close to the surface as possible where the bone quality is often the best. The HCS was always placed over a k-wire. The appropriate screw length was chosen to prevent interference with subsequent reduction of the reconstructed fragment into the fracture. An example is shown in Fig. 1. The screws were buried intra-osseous to not interfere with overlying implants or other fragments.

For fixation of a tibial eminence fragment, the screws were placed either antegrade (from the fragment toward distal) or retrograde (from distal into the fragment).

Once the HCS was in place and all the fragments reduced as anatomic as possible, fixation was completed with a buttress (locking) plate with rafting screws. Autologous bone graft from ipsilateral iliac crest or bone graft substitutes were used to fill large metaphyseal gaps caused by dis-impacting the articular fragments.

Postoperatively, a removable splint was applied for 10 days to allow wound healing. Patients were put on anticoagulant and antibiotics for 24–48 h. Depending on fracture pattern and fixation used, patients were allowed early range of motion on post-operative day one. No continuous passive motion was used. Patients were started on toe-touch weight-bearing for the first 6 weeks which was then increased to partial weight-bearing for another 6 weeks. Full weight-bearing was allowed at 3 months.

### Explanatory variables and outcomes

Clinical baseline variables retrieved from the electronic medical record included age, gender, mechanism of injury, Schatzker and AO/OTA fracture type [8, 9], and surgical treatments before the index procedure.

Surgical variables included fragment fixation type, operative approach, and number and length of the HCS, and their location. For presentation, we categorized the fragment fixation type into the following categories: (I) inter-fragmentary fixation of multiple, adjacent intra-articular fragments, (II) fixation of solitary intra-articular fragments (against an already reduced or non-fractured part of the tibial plateau), and (III) fixation of the tibial eminence. The location of fragments was described using the ten-column classification [10].

Clinical outcomes included (time to) union, reduction quality, arthrosis according to the Kellgren and Lawrence classification, range of motion (ROM), complications, and secondary surgeries. Union was determined by 3 out of 4 cortices having healed on plain radiographs and clinical assessment. In case of doubt of union, a dual energy CT scan with metal artifact reduction was obtained. Reduction quality was determined by measuring the maximal gap, step-off, and depression. Reductions were classified as anatomic (0 mm step-off, gap or depression), good (≤ 2 mm), fair (2–5 mm), or malreduction (> 5 mm) [11]. Depression was measured by drawing a line through the base of the tibial eminence parallel to the femoral condyles and then measuring the distance to the base of the tibial plateau. Arthrosis was assessed on the last available radiograph. The Kellgren and Lawrence classification system grades arthrosis on AP radiographs as 0 (none), 1 (doubtful), 2 (minimal), 3 (moderate), or 4 (severe), based on joint space narrowing, presence of osteophytes, sclerosis, and deformity [12].

Patient-reported outcome measures consisted of functional outcomes assessed with the Knee Injury and Osteoarthritis Outcome Score (KOOS) [13]. Outcome measures from patients that underwent TKA were recorded but excluded from statistical analysis.

### Statistical analysis

Categorical variables are presented as frequencies with percentages. Numerical variables are presented as medians with interquartile range (IQR). Missing data were accounted for by pairwise deletion (complete case analysis). Normality of the data was assessed by visual inspection of histograms. Explanatory variables are categorized for: follow-up duration (0–3, 3–6, 6–9, or > 9 years); injury mechanism (low or high energy), Schatzker type (unicondylar I–IV or bicondylar V–VI); surgical approach (single or dual incision); reduction quality (anatomical or non-anatomical), and arthrosis grade (0–1 or 2–4). In bivariate analysis, differences in KOOS scores are assessed using Kruskal–Wallis test or Mann–Whitney *U* test based on the number of categories of the explanatory variable (based on non-normal distribution the data). Data from the electronic medical record were collected using a Microsoft Excel spreadsheet. Patient-reported outcomes were collected using Castor EDC [14]. Significance was set at two-tailed alpha < 0.05. All statistical analyses were performed using SPSS, version 27.0 (SPSS Inc., Chicago, IL, USA).

## Results

### Clinical baseline and surgical variables (*n* = 29)

There were 15 males and 14 females with an average age of 46 years (IQR 37–57). According to the Schatzker classification, there were 8/29 (28%) Schatzker II fractures, 1/29 (3%) Schatzker III, 6/29 (21%) Schatzker IV, 7/29 (24%) Schatzker V, and 7/29 (24%) Schatzker VI [8]. According to the AO/OTA classification, there were 14/28 (50%) 41B3 fractures, 9/28 (32%) 41C1, 2/28 (7%) 41C2, and 4/28 (14%) 41C3 [9]. One Schatzker type IV fracture was a variant with an isolated posterocentral fragment and was not classified according to AO/OTA. All injuries were closed. The mechanisms of injury were 8 falls, 11 traffic accidents (7 bike, 2 scooter, 2 motorbike) and 5 skiing accidents. There were missing data for the mechanism of injury in 5 patients.

One patient was treated with revision osteo-synthesis due to an unfavorable result from ORIF in another country.

There were 9/29 (31%) fractures temporary stabilized with an external fixator. In 2/29 (7%) patients, compartment syndrome developed, and a fasciotomy was performed prior to ORIF.

According to our categorization, the HCS was used in 10/29 (34%) patients for inter-fragmentary fixation of intra-articular fragments, in 8/29 (28%) for fixation of a solitary, intra-articular fragment and in 8/29 (28%) for fixation of the tibial eminence. In 2/29 (7%) patients, the HCS was used for inter-fragmentary fixation of intra-articular fragments and fixation of the tibial eminence concomitantly. In the remaining patient, multiple HCS were used for fragments of the tibial tuberosity. Individual patients and their specific fragment fixation are detailed in Table 1.

**Table 1:** Characteristics and outcomes of individual patients and fragments

Patient	Age and sex	Schatzker/AO-OTA/side	Fixation type		Fragment(s) description	Number of screws (length)		Position of screw(s) through the fragment(s)	Reduction	ROM (months post-op)	Arthrosis grade (months post-op)	TKA	FU duration PROMs	KOOS symptoms and stiffness	KOOS pain	KOOS ADL	KOOS Sports	KOOS QOL
1	13 F	4/41B3.3/R	SF		Tibial eminence	2 (30 mm)		1 and 2: antegrade and anteromedial to posterior	Good	130–0-0 (152)	0 (151)		14 years	68	81	87	55	31
2	58 M	4^a^/L	SF		PC fragment	2 (n.a.)		1 and 2: posterosuperior to anteroinferior	Anatomic	120–0-0 (12)	0 (12)							
3	57 F	6/41C1.2/L	SF		ALC fragment	1 (n.a.)		1: anterolateral to posteromedial	Anatomic		1 (22)							
4	65 F	6/41C1.2/R	SF		Tibial eminence	1 (n.a.)		1 and 2: antegrade and anteromedial to posterior	Anatomic		2 (5)	Yes, after 1 year	13 years	96	83	82	30	88
5	64 F	2/41B3.1/L	SF		ALL fragment	1 (n.a.)		1: anterior to posterior	Anatomic		0 (3)							
6	47 F	6/41C2.3/R	SF		Tibial eminence	1 (n.a.)		1: retrograde and anterolateral to posteromedial	Anatomic	130–0-0 (17)	1 (17)		10 years	75	47	84	25	50
7	38 M	4/41B3.3 h/R	SF		Multiple fragments of the tibial tuberosity	4 (n.a.)		1 and 2: anterior to posterior 3 and 4: anteromedial to lateral and proximal	Anatomic	95–5-0 (8)	1 (40)		10 years	93	100	100	90	88
8	42 M	5/41C3.1/R	SF		Tibial eminence	2 (n.a.)		1: retrograde and anterior 2: antegrade and anteromedial to lateral	Anatomic	110–0-0 (12)	1 (12)		10 years	79	83	82	40	31
9	34 F	4/41B3.3 h/L	SF		Tibial eminence	2 (30 mm)		1 and 2: retrograde and anterolateral to posteromedial	Fair	140–0-5 (8) after additional surgeries in outside institution	2 (9)		9 years	71	78	97	40	50
10	37 M	4/41B3.3 h/R	SF		Tibial eminence	1 (30 mm)		1: percutaneous, antegrade and anteromedial to posteromedial	Anatomic	110–0-0 (3)	1 (3)		8 years	96	89	96	55	56
11	57 M	5/41C1.2/R	SF		Tibial eminence	3 (30 mm)		1: antegrade and anterior 2 and 3: retrograde and anteromedial to lateral	Fair	85–5-0 (24)	2 (24)		8 years	71	56	62	20	25
12	50 M	2/41B3.3f/L	SF		ALC fragment	1 (30 mm)		1: anterior to posterior and medial	Anatomic	110–0-0 (7)	1 (7)							
13	52 M	6/41C1.2/L	SF		ALC/ALC fragment	1 (30 mm)		1: anterolateral to posteromedial	Anatomic	110–0-0 (73)	2 (72)		6 years	43	72	91	100	0
14	64 F	2/41B3.1/L	IF		LC fragment + PL fragment	1 (30 mm)		1: anterior aspect of the LC fragment to posterior into the PL fragment and buried inside the tibial plateau	Anatomic	120–0-0 (3)	1 (3)							
15	49 M	2/41B3.1/L	IF		4 ALC and ALL fragments	2 (20 mm)		1 and 2: anterolateral aspect of the most ALL fragment to medial (1) and posteromedial (2)	Anatomic	100–0-5 (26)	3 (23)							
16	22 M	6/41C3.1/L	A: IF B: SF		A: Central fragment + large AL fragment B: Tibial eminence	A: 2 (30 mm) B: 1 (30 mm)		A: 1 and 2: medial aspect of the central fragment to lateral into the AL fragment B: 1: antegrade and anterolateral to posteromedial	Anatomic	130–0-0 (25)	2 (25)		5 years	68	69	74	55	44
17	46 M	2/41B3.1/L	IF		3 AC and ALC fragments + ALL fragment	3 (30 mm)		1: medial aspect of the AC fragment to lateral into the ALC and ALL fragments and buried inside the tibial plateau 2: anterior aspect the ALC fragment toward posterior and placed just subcortically 3: anterolateral aspect of the ALL fragment toward posteromedial, placed just subcortically and a plate is placed over the screw	Good^b^		3 (9)	Yes, after 1 year	5 years	86	100	93	70	63
18	30 F	2/41B3.1/R	SF		Osteotomy of PLL cortex	1 (30 mm)		1: posterolateral to anteromedial	Fair	130–0-0 (3)	0 (3)		4 years	100	100	100	100	100
19	44 F	2/41B3.3/R	IF		ALL/PLL fragment + ALC fragment	2 (24 mm)		1: lateral aspect of the PLL fragment to anteromedial into the ALC fragment 2: anterior aspect of the ALL fragment to posteromedial into the ALC fragment Both screws are placed just below the cortex and a plate is placed over the screws	Good	95–5-0 (8)	1 (3)		1 years	21	42	65	0	13
20	46 F	5/41C1.2/R	SF		PLC fragment	2 (28 mm)		1 and 2: from the anterolateral tibial plateau toward posteromedial into the PLC fragment and buried inside the tibial plateau	Good	100–0-0 (24)	3 (23)		4 years	32	47	32	5	13
21	63 F	5/41C3.1/L	IF		ALL/PLL fragment + ALC/PLC fragment	2 (30 and 26 mm)		1 and 2: anterior and lateral aspect of the PLL/ALL fragment to posteromedial into the ALC/PLC fragment, placed just below the cortex and a plate is placed over the screws	Anatomic	120–0-0 (14)	1 (3)		4 years	71	81	90	0	56
22	42 M	2/41B3.1/L	IF		ALC fragment + ALL/PLL fragment	1 (26 mm)		1: medial aspect of the ALC fragment to lateral into the ALL/PLL fragment, the screw tip is drilled just below the lateral cortex of the ALL/PLL fragment, and a plate is placed over the screw tip	Anatomic	130–0-0 (19)	0 (15)		4 years	71	89	85	35	56
23	44 F	6/41C2.2/L	IF		PC/PLC fragment + PLL fragment	1 (30 mm)		1: medial aspect of the PC/PLC fragment to lateral into the PLL fragment and buried inside the tibial plateau	Anatomic	95–10-0 (19)	2 (19)		4 years	25	33	28	10	19
24	34 M	5/41C1.2/R	SF		Tibial eminence	1 (30 mm)		1: antegrade and anterior to posterior	Anatomic	90–0-0 (39)	1 (39)		3 years	32	22	28	0	6
25	55 M	6/41C1.1/L	IF		PLC + ALC fragment	1 (20 mm)		1: posterior aspect of the PLC fragment to anterior into the ALC fragment and buried inside the tibial plateau	Malreduction	120–0-0 (9)	3 (20)		3 years	54	44	44	20	38
26	52 F	5/41C3/L	A: IF B: SF		A: ALC/ALL fragment + PLL fragment B: Tibial eminence	A: 2 (30 mm) B: 2 (28 mm)		A 1: from the anterolateral aspect of the ALC/ALL fragment to posterior into the PLL fragment and placed just below the cortex 2: from the centeromedial aspect of the ALC/ALL fragment to posterolateral into the PLL fragment, buried inside the tibial plateau and the tip is placed just below the cortex of the PLL fragment B: 1 and 2 retrograde and anterolateral to posteromedial	Fair	100–10-0 (16)	1 (16)		2 years	50	61	85	15	25
27	33 M	2/41B3.1/L	IF		Multiple ALC, ALL and PLC fragments	2 (30 mm)		1 and 2: anterolateral aspect of the ALL fragment to medial and posteromedial, respectively, placed just below the cortex and a plate is placed over screw 2	Anatomic	100–0-0 (3)	0 (3)		2 years	93	89	96	85	63
28	67 M	4/41B3.3 h/L		IF		ALC fragment + AC fragment	1 (30 mm)	1: posterolateral aspect of ALC fragment to anteromedial into the AC fragment and buried inside the tibial plateau	Lost to follow-up (outside institution)									
29	37 F	5/41C1.2/L		SF		PMC fragment	1 (n.a.)	1: posterior to anterior	Anatomic		0 (70)		14 years	50	44	49	20	19

Blank cells indicate missing outcomes. Fragments are classified using the ten-segment classification [10]

*SF* single fragment, *IF* interfragmentary fixation, *ALL* antero-latero-lateral, *ALC* antero-latero-central, *AC* antero-central, *PLL* postero-latero-lateral, *PLC* postero-latero-central, *PC* postero-central, *PMC* postero-medio-central, *n.a.* not available, *ROM* range of motion, *FU* follow-up, *TKA* total knee arthroplasty, *PROM* patient-reported outcome measure

^a^Isolated postero-central fragment

^b^The lateral plateau later collapsed in an acute moment (4 months after the index procedure)

### Clinical outcome measures (*n* = 28)

Union was achieved in all patients at an average of 3 months (IQR 2.7–3.3). Reduction was graded as anatomic (19) or good (4) in 23/28 (82%) of cases, fair in 4/28 (14%), and as a malreduction in 1/28 (4%) on radiographs.

According to the Kellgren and Lawrence classification, arthrosis was grade 0 in 7/28 (25%) patients, grade 1 in 11/28 (39%), grade 2 in 6/28 (21%), and grade 3 in 4/28 (14%).

Range of motion was described for 23/28 (82%) patients. Median flexion was 110 degrees (IQR 100–130), and five patients had an extension deficit of 5 (*n* = 3) and 10 (*n* = 2) degrees.

Complications were seen in 7/28 (25%) patients. There were two deep and one superficial surgical site infections (SSI) [15]. Two patients with a deep SSI were successfully treated with revision fixation, debridement, and antibiotics and with debridement and antibiotics alone, respectively. The latter of these patients developed a footdrop, presumably due to peroneal nerve traction injury. One patient had a superficial SSI due to a remaining stitch which healed well without antibiotics. The patient was later diagnosed with a malunion (valgus axis) of the lateral plateau for which revision surgery was performed 7 months after the index procedure. The patient healed with good alignment 3 months later. Two other patients had a systemic complication: dyspnea, minor cardiac ischemia, and an allergic rash to antibiotics (*n* = 1), and deep venous thrombosis (*n* = 1). All complications resolved, except the peroneal nerve traction injury.

Two patients (7%) had a complication directly related to the HCS. One patient (patient 15) had inter-fragmentary fixation of 4 fragments for a Schatzker type II fracture and presented 22 months after the procedure with pain in the knee. A CT scan showed that one of the HCS had migrated behind the femoral head. There was no loss of reduction. All materials were removed in the operating theater, but the HCS could not be retrieved. Afterward the complaints improved, and the patient could walk 2 km without pain, indicating that the migrated screw was not the cause of the pain. In retrospect, the screw was not optimally placed within the subchondral bone as noted on a post-operative CT-scan.

One patient (patient 17) had inter-fragmentary fixation of 3 fragments for a Schatzker type II fracture. He admitted to mis-stepping several times within 4 months of the initial procedure. The patient experienced an ‘acute’ moment while mis-stepping in a pit while carrying a load which caused a collapse of the centrolateral plateau and migration of one HCS laterally into the soft tissue. The migrated HCS and one other HCS were removed. Five months later, an additional k-wire was removed. He underwent TKA within one year of the index surgery. At latest follow-up, he was doing well, including downhill skiing.

Other secondary surgeries consisted of hardware removal in 9/28 (32%) patients due to pain caused by the osteo-synthesis material. In 5 of these, at least one HCS was left in place because they were completely intra-osseous. One patient underwent surgery for heterotopic ossification around the patellar tendon and the medial ligaments. This may have been caused by excessive bone formation after demineralized bone matrix treatment [16]. Three patients had an arthroscopic procedure for arthrofibrosis (*n* = 2) and debridement of a small lateral meniscus tear (*n* = 1). One of these patients received an additional surgery in an outside institution to increase range of motion by arthrolysis. One additional patient underwent TKA for symptomatic arthrosis within 1 year after the index procedure.

### Patient-reported outcome measures (*n* = 20)

The median KOOS score for symptoms and stiffness was 69 points (IQR 45–78), for pain 71 (IQR 45–88), for activities of daily living (ADL) 85 (IQR 52–95), for sports 30 (IQR 11–55), and for quality of life (QOL) 34 (IQR 19–56). The KOOS sub-scores are presented for categories of follow-up duration, injury mechanism, Schatzker type, surgical approach, reduction quality, and arthrosis grade in Table 2. Patients with unicondylar (Schatzker I–IV) reported better scores on all outcomes when compared to patients with bicondylar fractures (V–VI). This was a statistically significant difference for pain (88.9 vs. 51.4; *p* = 0.005), ADL (95.6 vs. 67.6; *p* = 0.002), sports (55.0 vs. 20.0; *p* = 0.047)*,* QOL (56.3 vs. 25.0; *p* = 0.012), and overall KOOS (72.9 vs. 47.0; *p* = 0.002). Similarly, patients that underwent a single-incision surgical approach (compared to dual incision) had significantly better outcomes for pain (88.9 vs. 61.1; *p* = 0.042), QOL (62.5 vs. 31.3; *p* = 0.033), and overall KOOS (85.0 vs. 56.2; *p* = 0.033). No significant differences in scores were observed when comparing categories of follow-up duration and injury mechanism. Although patients with anatomical reduction (compared to non-anatomical reduction) showed higher scores on all subscores, this did not reach significance.

**Table 2:** Median KOOS subscores and overall KOOS scores for categories follow-up duration, injury mechanism, Schatzker type, surgical approach, reduction quality, and arthrosis grade

	KOOS symptoms and stiffness (*n*)	KOOS pain (*n*)	KOOS ADL (*n*)	KOOS sports (*n*)	KOOS QOL (*n*)	KOOS overall (*n*)
Follow-up duration (*n* = 20)						
0–3 years	51.8 (4)	52.8 (4)	75.0 (4)	17.5 (4)	31.3 (4)	43.6 (4)
3–6 years	67.8 (7)	69.4 (7)	73.5 (7)	10.0 (7)	43.8 (7)	59.6 (7)
6–9 years	71.4 (3)	72.2 (3)	91.2 (4)	55.0 (3)	25.0 (3)	61.3 (3)
> 9 years	73.0 (6)	79.2 (6)	85.3 (6)	40.0 (6)	40.6 (6)	63.7 (6)
Injury mechanism (*n* = 18)^a^						
Low energy	69.6 (10)	80.6 (10)	85.3 (10)	25.0 (10)	31.3 (10)	61.3 (10)
High energy	69.4 (8)	70.8 (8)	82.4 (8)	47.5 (8)	40.6 (8)	61.6 (8)
Schatzker (*n* = 20)						
I–IV (unicondylar)	82.1 (8)	**88.9 (8)**	**95.6 (8)**	**55.0 (8)**	**56.3 (8)**	**72.9 (8)**
V–VI (bicondylar)	51.8 (12)	**51.4 (12)**	**67.6 (12)**	**20.0 (12)**	**25.0 (12)**	**47.0 (12)**
*p* value		0.005	0.002	0.047	0.012	0.002
Approach (*n* = 20)						
Single	92.9 (5)	**88.9 (5)**	95.6 (5)	85.0 (5)	**62.5 (5)**	**85.0 (5)**
Dual	67.9 (15)	**61.1 (15)**	82.4 (15)	20.0 (15)	**31.3 (15)**	**56.2 (15)**
*p* value		0.042			0.033	0.033
Reduction (*n* = 20)						
Anatomical	71.4 (12)	76.4 (12)	84.6 (12)	37.5 (12)	46.9 (12)	61.6 (12)
Non-anatomical	60.7 (8)	58.3 (8)	75.0 (8)	20.0 (8)	28.1 (8)	47.0 (8)
Arthrosis (*n* = 20)						
No (grade 0–1)	71.4 (13)	80.6 (13)	85.3 (13)	35.0 (13)	50.0 (13)	63.1
Yes (grade 2–4)	53.6 (7)	55.6 (7)	61.8 (7)	20.0 (7)	25.0 (7)	46.8 (7)

Bold indicates statistical significance

^a^In 2 patients injury mechanism was unknown

## Discussion

The main objective in the surgical management of a tibial plateau fracture is anatomic restoration and stable fixation that allows early motion. In this report, we present a series of 29 patients with a comminuted tibial plateau fracture that were treated with open reduction and internal fixation using plates and screws with the addition of fully threaded headless cannulated compression screws for a “two-level fixation”.

Stable anatomic reduction of the tibial plateau can be challenging when there are more than 2–3 fragments without a cortical border. The lateral plateau is more prone to fragmentation and impaction due to its convex shape, as opposed to the medial tibial plateau which is concave [17]. Therefore, an axial load on the knee joint causes a multi-fragmentary lateral depression type fracture. We were the first to map tibial plateau fractures with CT images and identified a lateral split fragment with or without comminution (defined as fragments smaller than 1.0 cm^2^ in size) as one of four main features of tibial plateau fractures [18].

For this fracture pattern, standard treatment includes the use of a rafting construct to provide axial support [19]. Authors have suggested different construct variations to better support small fragments. Yoon et al. described the “inside out” technique, in which articular fragments are fixated with a k-wire that is inserted from lateral to medial and then retrieved from the medial side until the k-wire is on par with the lateral side of the fragment, after which the lateral split fragment is reduced, and the k-wires are advanced back to the lateral side [20].

As an alternative, Giordano et al. used a flattened one-third tubular plate that was placed in the subchondral bone after reduction of depressed fragments to treat four tibial plateau fractures with articular depression [21]. All four patients healed without a significant loss of reduction.

Similar to our technique, Reul et al. used free subchondral screws in 23 patients with depressed intra-articular fragments of the lateral plateau [22]. They performed an arthrotomy and osteotomy of the lateral tibial condyle after which the depressed fragment was reduced under direct visualization and fixated with one or more 2.7 mm stable angle screws. These were placed in the subchondral bone and intra-osseous. Full ROM was achieved in 10/23 (43%) patients, and 3/23 (13%) patients’ flexion was reduced by 30 degrees or more. 58% of patients’ reduction was suboptimal with a step-off of more than 2 mm and a suboptimal alignment based on post-operative CT scans.

In this report, we introduced the use of HCS to provide additional support for small fragments in comminuted tibial plateau fractures. Our standard to fragment fixation includes the use of k-wires or lag screws and the use of a rafting construct for support. The use of the HCS was indicated in fractures that presented with (multiple) small, intra-articular or tibial eminence fragment(s) that could not be sufficiently reduced with the use of lag screws (due to the absence of a cortical border) or k-wires (due to insufficient compression), or that needed additional support complementary to the rafting screw construct (due to an inadequate purchase of bone stock of the fragment). Although there were no specific contra-indications to the use of the HCS, a relative contraindication might be the use of HCS in elderly patients as these are prone to secondary a loss of fixation, especially in case of fractures with extensive fragmentation [23]. Because the HCS are buried intraosseous, removal during revision surgeries might propose problems. In our 15-year experience, we have not encountered this problem. This limitation is also relevant in patients that require TKA. In our experience, one patient that underwent TKA had a HCS in place at the moment of surgery. As this procedure was performed in an outside institution, we do not know if this caused any problems. Difficult removal could also be problematic in case of a deep infection. We therefore urge surgeons to be meticulous in their approach to prevent infectious complications. It should be noted that k-wires are easier to remove than HCS, which could circumvent some of the limitations of the HCS mentioned above. However, k-wires do not have the benefit of providing inter-fragmentary fixation and HCS have biomechanical advantages over k-wires [24].

**Fig. 2:**
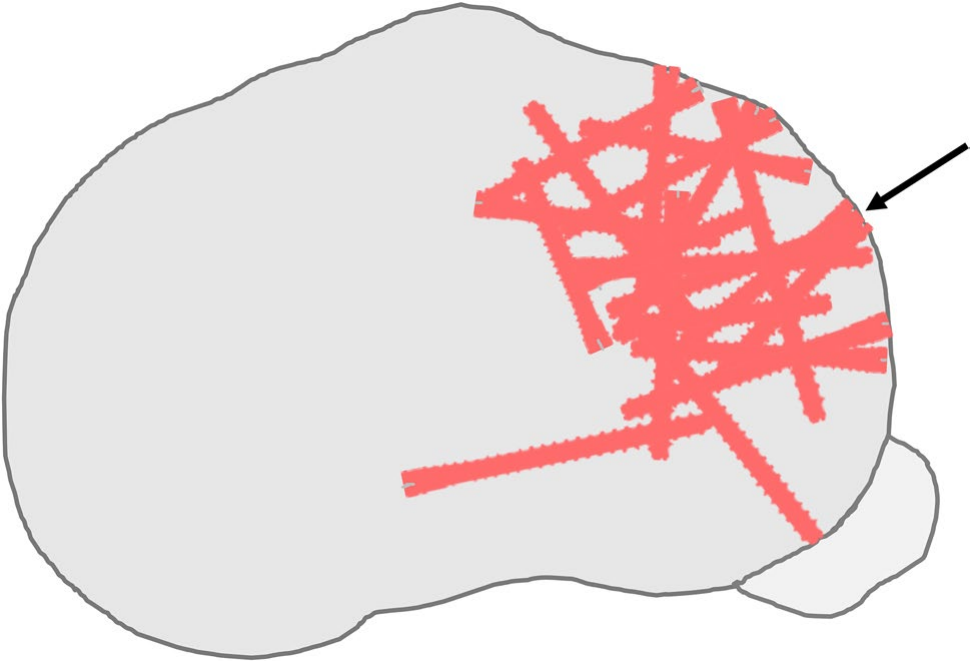
Superimposition of the HCS used for interfragmentary fixation in the lateral tibia plateau. The arrow indicates the two screws that migrated

In our series, we found the HCS to be very helpful for inter-fragmentary fixation of intra-articular fragments in the lateral plateau when standard fixation was deemed insufficient (Fig. 2). The benefits of the HCS are the following: first, the screws can be introduced from multiple directions, which allows for an optimal purchase of bone stock. Second, the head of the screw can be buried inside the plateau (intra-osseous), which enables freedom for subsequent placement of overlying plates and other fragments (“two-level” fixation) and is less likely to cause complaints. Third, it is possible to perform inter-fragmentary fixation on a side table under direct visual control. The variable pitch of the HCS provides compression between these fragments that can then be reduced into the fracture as a whole. To optimize the amount of inter-fragmentary compression, one should maximize screw length and place the center of the screw perpendicular to the fracture line [25]. Fourth, as they are titanium, they cause less artifact when performing MRI to evaluate post-traumatic changes of the cartilage, menisci, and cruciate ligaments.

The patient-reported functional outcomes of our series were compared to the existing literature. Jansen et al. evaluated 22 patients with intra-articular AO/OTA type C fractures after a mean follow-up of 67 months [26]. They report a mean KOOS score of 68 points (our series median: 60 points). Henkelmann et al. evaluated 246 patients with a AO type B and C tibial plateau fractures (without post-operative SSI) after a mean follow-up of 78.9 months [27]. They report mean KOOS subscores for pain of 75 (our series median: 71), symptoms and stiffness 75 (our series median: 69), ADL 81 (our series median: 85), sports 41 (our series median: 30), and QOL 56 (our series median: 34). Dreumel et al. evaluated 71 patients with a tibial plateau fracture treated with ORIF after a mean of 6 years [28]. They report median KOOS subscores for pain of 90, symptoms and stiffness of 91, ADL of 90, sports of 73, and QOL of 75. Timmers et al. assessed 82 patients with Schatzker type I to VI fractures treated with ORIF, and report similar KOOS subscores for symptoms and stiffness, pain, and ADL, but better subscores on sports (50 vs. our series: 30) and QOL (55 vs. our series: 34) [29]. An important difference between these studies and our series is that we used a dual incision approach with double plating in 52% of patients (compared to Jansen et al.: 14% [26], Henkelmann.: 14% [27], Timmers et al.: 3% [29]), which might explain the difference in scores: bivariate analysis in our series demonstrated that patients with bicondylar fractures (and consequently dual incision treatment) reported significantly lower functional outcomes. Another factor that might influence these results is indication bias; we only used HCS for fractures with comminuted, small, and intra-articular fragments in which reduction with standard implants was deemed insufficient. Lastly, it should be noted that all subscores of the KOOS in our series demonstrated substantially large interquartile ranges, depicting that there was a considerable difference between the outcomes reported by our patients.

The most feared short-term complication after tibial plateau fracture surgery is a SSI. These occur in approximately 10% of cases (range 2.6–45.0%; of which deep: 6.4%, superficial: 3.6%), and are associated with worse clinical and patient-reported outcomes [27, 30]. In our series, the rate of deep and superficial SSI was 7 and 4%, respectively, and all infections resolved with appropriate treatment. Infection risk can be mitigated by adequate timing of the surgery and careful soft tissue management. Other complications associated with tibial plateau fractures are malunions and systemic complications such as deep vein thrombosis. These complications only occurred incidentally in our series.

The most important long-term complication after a tibial plateau fracture is posttraumatic arthrosis. Important determinants for the development of arthrosis include alignment and articular congruity [31, 32]. In our series, 35% of patients had at least minimal signs of arthrosis at the end of clinical follow-up. This is in range with the literature. In a previous report of 109 tibial plateau fractures from our department, 31% of patients had developed secondary arthrosis after operative treatment [31]. Manidakis et al. found evidence of arthrosis in 26.4% of patients [33]. For Schatzker type V and VI fractures, this was as a high as 58%. Jansen et al. reported signs of arthrosis in 70% [26]. In our series, patients with arthrosis had considerably worse scores on all subscales of the KOOS, but this did not reach statistical significance. Despite the presence of arthrosis, we only know of 2/28 (7%) patients that underwent TKA. In one patient because of symptomatic arthrosis and in one patient due to a significant collapse of the lateral plateau after a misstep. This rate of TKA after operative treatment of tibial plateau fractures is within range of previous literature [28, 29, 33, 34].

The present study has several limitations. First, the retrospective design is associated with methodological drawbacks, such as the lack of predefined follow-up variables and standardized assessment of outcomes. For example, we do not routinely follow patients with CT scans to limit radiation exposure and as a consequence, we relied on standard radiographs to determine fracture reduction quality. Radiographs are less sensitive to detect reduction defects, which was also seen in two of our cases [35]. Moreover, we did not perform long leg standing radiographs and were therefore not able to determine alignment. Nevertheless, we were able to collect relevant clinical outcome variables from the electronic patient records and we were able to retrieve (long-term) functional outcomes of 76% of patients, which is comparable to previous studies [28, 29]. Second, we did not include a matched cohort to compare the use of HCS to standard fixation techniques for fragments (i.e., k-wires or lag screws). However, a case–control design would not provide an appropriate comparison due to selection bias: we used HCS only in fractures were standard fixation was deemed insufficient. Therefore, a comparison between these techniques would be inherently biased by the fracture pattern. Third, there was no single standardized surgical protocol as the screws were used to treat a variety of fragment types. However, we also believe that this variety displays the versatility to fixate fragments with these screws. Fourth, all patients were treated by a single surgeon in a single institution, which questions the generalizability to the overall population. However, the procedure is a relatively simple variation of standard treatment and can therefore easily be used by other surgeons as well. Lastly, comparing outcomes of treatment of tibial plateau fractures is difficult due to the variation in fracture patterns, treatments, and follow-up protocols. Therefore, we cannot make claims on the superiority of our approach to comminuted tibial plateau fractures.

In conclusion, the use of fully threaded headless cannulated compression screws is a simple and helpful addition in the treatment of comminuted tibial plateau fractures in which fixation of intra-articular fragments with k-wires or lag screws is deemed insufficient. The screws can be used for two-level, (inter-fragmentary) fixation of intra-articular fragments and for fixation of the tibial eminence.
